# Supplementary material for: Discovery and application of insertion-deletion (INDEL) polymorphisms for QTL mapping of early life-history traits in Atlantic salmon
Source: BMC Genomics. 2010 Mar 8;11:156. doi: 10.1186/1471-2164-11-156 (PMC2838853; doi:10.1186/1471-2164-11-156)
Supplement: Additional file 2 — Information on developed 76 locus single-run INDEL panel in Atlantic salmon. Information on fluorescence labeling, primer concentrations, PCR pooling and links to alignments, INDEL motifs and GENESCAN (Burge and Karlin 1997) predictions of genes/exons are available in html format. [file 1471-2164-11-156-S2.ZIP › Additionalfile2/snpsummary4493.html]

```
Cluster 1054 Contig 1

prev  Summary    Contig List  next
```

Size of Consensus sequence = 1931

Number of sequences = 17

Minimum redundancy = 6

Key

A gi|117519446|gb|EG851174.1|EG851174 EST\_ssal\_eve\_8449 ssaleve thyroid Salmo salar cDNA Salmo salar cDNA clone ssal\_eve\_510\_167\_rev 5', mRNA sequence  
B gi|24206577|gb|BU965830.1|BU965830 ssalmna004096 muscle\_skin Salmo salar cDNA, mRNA sequence  
C gi|89875330|gb|DY731453.1|DY731453 EST\_ssal\_rgb2\_87192 ssalrgb2 mixed\_tissue Salmo salar cDNA Salmo salar cDNA clone ssal\_rgb2\_642\_254\_fwd 3', mRNA sequence  
D gi|117424855|gb|EG757079.1|EG757079 EST\_ssal\_sjb\_5775 ssalsjb mixed\_tissue Salmo salar cDNA Salmo salar cDNA clone ssal\_sjb\_013\_172\_fwd 3', mRNA sequence  
E gi|117519448|gb|EG851176.1|EG851176 EST\_ssal\_eve\_8450 ssaleve thyroid Salmo salar cDNA Salmo salar cDNA clone ssal\_eve\_510\_167\_fwd 3', mRNA sequence  
F gi|24390316|gb|CA060073.1|CA060073 ssalrga515167 mixed\_tissue Salmo salar cDNA, mRNA sequence  
G gi|89842301|gb|DY699339.1|DY699339 EST\_ssal\_rgb2\_55078 ssalrgb2 mixed\_tissue Salmo salar cDNA Salmo salar cDNA clone ssal\_rgb2\_589\_005\_fwd 3', mRNA sequence  
H gi|24382651|gb|CA052408.1|CA052408 ssalrgb527200 mixed\_tissue Salmo salar cDNA, mRNA sequence  
I gi|24393407|gb|CA063148.1|CA063148 ssalrgb509347 mixed\_tissue Salmo salar cDNA, mRNA sequence  
J gi|89840459|gb|DY698297.1|DY698297 EST\_ssal\_rgb2\_54036 ssalrgb2 mixed\_tissue Salmo salar cDNA Salmo salar cDNA clone ssal\_rgb2\_587\_142\_fwd 3', mRNA sequence  
K gi|89859622|gb|DY715745.1|DY715745 EST\_ssal\_rgb2\_71485 ssalrgb2 mixed\_tissue Salmo salar cDNA Salmo salar cDNA clone ssal\_rgb2\_615\_327\_fwd 3', mRNA sequence  
L gi|24352561|gb|CA046391.1|CA046391 ssalbrh007028 head Salmo salar cDNA, mRNA sequence  
M gi|89842485|gb|DY699452.1|DY699452 EST\_ssal\_rgb2\_55191 ssalrgb2 mixed\_tissue Salmo salar cDNA Salmo salar cDNA clone ssal\_rgb2\_589\_069\_fwd 3', mRNA sequence  
N gi|85049269|gb|DW577447.1|DW577447 EST\_ssal\_rgb2\_41866 rgb2 Salmo salar cDNA clone ssal\_rgb2\_567\_332\_fwd 3', mRNA sequence  
O gi|89849255|gb|DY705378.1|DY705378 EST\_ssal\_rgb2\_61117 ssalrgb2 mixed\_tissue Salmo salar cDNA Salmo salar cDNA clone ssal\_rgb2\_598\_139\_fwd 3', mRNA sequence  
P gi|29326664|gb|CB515438.1|CB515438 ssalrgb554051 mixed\_tissue Salmo salar cDNA, mRNA sequence  
Q gi|29328093|gb|CB516867.1|CB516867 ssalrgb555179 mixed\_tissue Salmo salar cDNA, mRNA sequence

2 SNPs detected

A B C D E F G H I J K L M N O P Q  cosegregation weighted

1210 . . - - - A - A A - A - A . - A A   2/2 82.35
1211 . . - - - C - C C - C - C . - C C   2/2 82.35
